# Short-term follow-up of chagasic patients after benznidazole treatment using multiple serological markers

**DOI:** 10.1186/1471-2334-11-206

**Published:** 2011-07-31

**Authors:** Ana Fernández-Villegas, María Jesús Pinazo, Concepción Marañón, M Carmen Thomas, Elizabeth Posada, Bartolomé Carrilero, Manuel Segovia, Joaquim Gascon, Manuel C López

**Affiliations:** 1 Departamento de Biología Molecular. Instituto de Parasitología y Biomedicina López Neyra - Consejo Superior de Investigaciones Científicas (IPBLN-CSIC). Parque Tecnológico de Ciencias de la Salud - Avda. del Conocimiento s/n. 18100-Granada, Spain; 2 Barcelona Centre for International Health Research (CRESIB), Hospital Clínic of Barcelona, c/Rosselló 132, 08036-Barcelona, Spain; 3 Servicio de Microbiología (Unidad Regional de Medicina Tropical), Hospital Virgen de la Arrixaca. Carretera Madrid-Cartagena s/n, El Palmar, 30120-Murcia, Spain

## Abstract

**Background:**

Conventional serological tests, using total soluble proteins or a cocktail of recombinant proteins from *T. cruzi *as antigens, are highly sensitive for Chagas disease diagnosis. This type of tests, however, does not seem to be reliable tools for short- and medium-term monitoring of the evolution of patients after antiparasitic treatment. The aim of the present study was to search for immunological markers that could be altered in the sera from Chagas disease patients after benznidazole treatment, and therefore have a potential predictive diagnostic value.

**Methods:**

We analyzed the reactivity of sera from chagasic patients during different clinical phases of the disease against a series of immunodominant antigens, known as KMP11, PFR2, HSP70 and Tgp63. The reactivity of the sera from 46 adult Chronic Chagas disease patients living in a non-endemic country without vector transmission of *T. cruzi *(15 patients in the indeterminate stage, 16 in the cardiomiopathy stage and 16 in the digestive stage) and 22 control sera from non-infected subjects was analyzed. We also analyzed the response dynamics of sera from those patients who had been treated with benznidazole.

**Results:**

Regardless of the stage of the sickness, the sera from chagasic patients reacted against KMP11, HSP70, PFR2 and Tgp63 recombinant proteins with statistical significance relative to the reactivity against the same antigens by the sera from healthy donors, patients with autoimmune diseases or patients suffering from tuberculosis, leprosy or malaria. Shortly after benznidazole treatment, a statistically significant decrease in reactivity against KMP11, HSP70 and PFR2 was observed (six or nine month). It was also observed that, following benznidazole treatment, the differential reactivity against these antigens co-relates with the clinical status of the patients.

**Conclusions:**

The recombinant antigens KMP11, PFR2, Tgp63 and HSP70 are recognized by Chagas disease patients' sera at any clinical stage of the disease. Shortly after benznidazole treatment, a drop in reactivity against three of these antigens is produced in an antigen-specific manner. Most likely, analysis of the reactivity against these recombinant antigens may be useful for monitoring the effectiveness of benznidazole treatment.

## Background

Chagas disease or American trypanosomiasis is a complex anthropozoonosis caused by the flagellate protozoan parasite *Trypanosoma cruzi*. This sickness affects around 8 million people in Latin-America despite the intensive programs implemented to control the illness-transmitting vectors [1-3]. In addition, the increasing number of migrants from Latin-American countries has globally spread the *T. cruzi *infection to non-endemic areas [4,5]. Nowadays, other ways of infection such as congenital transmission, blood transfusion and organ transplantation are becoming prevalent and relevant from a public health point of view in both endemic and non-endemic countries [6].

The disease passes through various different clinical stages. The parasite can be visualized in the blood stream during the acute stage and eventually detected by PCR in the chronic stages of the disease. In absence of treatment, the acute phase is followed by an indeterminate stage in which the parasites are present into specific tissues [7]. In 30% of patients, the infection leads to a symptomatic chronic phase. Despite low mortality during this symptomatic stage, serious cardiac and/or digestive alterations are present [7,8]. Arrhythmias, electrocardiographic abnormalities together with cardiomegaly and/or systolic dysfunction may appear when there is cardiac damage [9,10]. Megaesophagus or megacolon are indicative of gastrointestinal damage and, although these clinical manifestations are usually not highly severe, they are associated to morbidity [11].

Anti-trypanosomal treatment is strongly recommended for all cases of the acute, congenital and reactivated infection of *T. cruzi*, and for the treatment of young chronic patients [3]. However, its efficacy for treatment of adult patients in the chronic phase of the disease is under consideration [12,13]. New drugs are being currently examined, some now in the advanced stages of development [14].

At present, the most widely used serological tests for Chagas disease diagnosis are based on homogenates of total parasite proteins or combinations of recombinant proteins as antigens [15-17]. Although all these techniques are very sensitive for the diagnosis of Chagas disease [18], the evaluation of the evolution of the patients under and following treatment is ambiguous since some *T. cruzi *antibodies are long lasting [19] and a significant seroconversion occurs only several years post treatment [11,20]. Thus, conventional serological tests are not useful for short- and medium-term post-treatment monitoring as they do not allow early recognition of a therapeutic failure [21-23]. Consequently, reliable tools for the evaluation of the therapeutic efficacy of the drugs are needed.

The aim of the present study was to search for immunological markers, against which the reactivity of sera from Chagas disease patients could be modified by benznidazole treatment, thus providing potential predictive diagnostic value.

## Methods

### Human sera

Serum samples from 46 adult Chagas disease patients and 22 control sera from healthy adult donors (HD) were collected at the Hospital Clínic in Barcelona and Hospital Virgen de la Arrixaca in Murcia (Spain). All patients and healthy donors came from endemic areas and were Spanish residents in whom *T. cruzi *reinfection does not occur (Table 1). The patients included in the study had never received benznidazole/nifurtimox treatment.

**Table 1:** Characteristics of the population under study

**SUBJECTS **^**a**^	MEAN AGE IN YEARS (range)	MALE (%)	FEMALE (%)	ORIGIN (%)
**HD (n = 22)**	31.5 (18-54)	14 (63.6)	8 (36.4)	15 Bolivia (70); 7 Ecuador (30)

**IND (n = 15)**	33.5 (22-56)	14 (93.3)	1 (6.67)	13 Bolivia (86.6); 1 Brasil (6.7); 1 Paraguay (6.7)

**CCC (n = 16)**	44.1 (27-68)	9 (56.3)	7 (43.7)	14 Bolivia (87.5); 1 Brasil (6.3); 1 Argentina (6.3)

**DIG (n = 15)**	39.1 (24-56)	10 (66.6)	5 (33.4)	14 Bolivia (93.3); 1 Argentina (6,7)

^a ^HD: Healthy donors. IND: Chronic chagasic patients at indeterminate clinical form of the disease. CCC: Chronic cardiac chagasic patients. DIG: Chronic chagasic patients with abnormalities in the gastrointestinal track

Following WHO criteria, Chagas disease diagnosis was made using two different commercial serological tests (ELISA-Bioelisa Chagas, Biokit (Barcelona, Spain) and IFI-Inmunofluor Chagas, Biocientífica, Argentina) previous to inclusion. All patients were at the chronic stage of the disease and were classified into three main categories. Chagasic patients were considered at the Indeterminate stage (IND, n = 15) when there was no evidence of cardiac or gastrointestinal disorders; the Chronic Chagas Cardiomiopathy stage (CCC, n = 16) was catalogued into G1 to G3 stages of Kuschnir classification [24] according to clinical criteria and radiological, electrocardiographic and echocardiography analyses; the digestive stage (DIG, n = 15) was established when mega-esophagus and/or megacolon abnormalities in the gastrointestinal tract were detected by barium enema and esophagogram. All IND and CCC and 10 DIG chagasic patients were treated with benznidazole (5 mg/kg/day for 60 days) and clinically followed up during the period of this study. No changes in the clinical status of the patients were observed during this follow-up period.

Blood samples were collected prior to treatment administration (T0) and at regular time intervals of three (T1), six (T2) and nine (T3) months post-treatment. Aliquots of the sera were stored at -80°C in 50% glycerol.

Serum samples from patients with different autoimmune diseases or patients suffering an infectious disease other than Chagas disease were also included in this study. Sera were collected from 15 patients suffering autoimmune diseases from non-endemic areas: Systemic Lupus Erythematosus (SLE) (n = 6), celiac disease (n = 4) and rheumatoid arthritis (n = 5). Sera were collected from Hospital de la Santa Creu i Sant Pau (Barcelona) as well as 24 serum samples from patients suffering leprosy (n = 8), tuberculosis (n = 8) and malaria (n = 8) from the Institute of Tropical Medicine (Caracas-Venezuela).

### Ethical considerations

The protocols were approved by the Ethical Committees at Hospital Clínic, Hospital Virgen de la Arrixaca and Consejo Superior de Investigaciones Científicas (Spain). A signed informed consent was obtained from all individuals prior to their inclusion in the study.

### Gene cloning and protein purification

The DNA sequence coding for the gp63 protein fragment (Tgp63) [25] was amplified by PCR using *Leishmania infantum *genomic DNA as template and Q5'gp63Li (5'CAAGGGTACCGGAACGACCTGCCAG 3') and Q3'gp63Li (5'CTGAAAGCTTACCCCGGCCCCACG 3') primers which bear *Kpn*I and *Hind*III restriction sites, respectively. The 483 nucleotide fragment coding for the highly conserved region of the protein which is implicated in the molecular interaction with the macrophage surface molecules [26] was digested with *Kpn*I and *Hind*III enzymes and subcloned into the pQE30 plasmid (Qiagen, California, USA) digested with the same enzymes.

Tgp63 recombinant protein was overexpressed in the *Escherichia coli *M15 strain by addition of 0.1 mM isopropyl-beta-D-thiogalactopyranoside (IPTG) and grown at 37°C for 3 h. Proteins were solubilized in resuspension buffer (0.3 M NaCl and 50 mM NaH_2_PO_4_, 1 mM phenyl methyl sulfonyl fluoride, pH 8) by sonication. The Tgp63 protein was bound overnight to the Ni^2+ ^NTA resin (Qiagen, California, USA) at 4°C with gentle shaking. After extensive washing with resuspension buffer at pH from 8 to 6, the Tgp63 protein was subsequently eluted with the same buffer at pH 4-5. *T. cruzi *KMP11 [27], HSP70 [28]. PFR2 [29] recombinant proteins were overexpressed in bacteria and purified as described previously.

To obtain soluble total protein extract (S*Tc*A), *T. cruzi *trypomastigote forms (Y strain) obtained from *T. cruzi *infected mice were used to infect monolayers of LLC-MK2 fibroblast cells at parasite:cell ratio of 5:1 for 12 hours. Cell cultures were washed to remove the parasites that were not able to infect cells and incubated at 37°C in an atmosphere containing 5% CO_2_. Amastigote/trypomastigote parasites (ratio 3/1) were recovered from infected-culture supernatants and washed in PBS. Parasites were resuspended in lysis buffer (50 mM Tris-HCl at pH 7.4, 0.05% NP-40, 50 mM NaCl, 1 mM PMSF, 1 μg/mL leupeptin) and sonicated. Soluble protein extracts were obtained after centrifugation at 10,000 rpm for 20 min at 4°C.

### ELISA measurement

ELISA tests were carried out as described in Thomas et al. [30]. Briefly, ELISA 8-well strips (Nunc, Roskilde, Denmark) were coated with 0.5 μg of protein and stored in a dry atmosphere at -20°C until use. Subsequently, the wells were washed twice with 200 μL of PBS-0.05% Tween-20 and incubated for one hour with blocking solution (5% nonfat dried milk powder in PBS). Afterwards, the antigen-coated wells were incubated with patient sera at 1/800 dilution (for HSP70, PFR2 and Tgp63), 1/3,200 (for KMP11), and 1/6,400 for S*Tc*A, for two hours at 37°C. Selection of serum dilutions was established for individual antigens using a pool of sera from 6 patients with Chagas disease who lived in endemic areas. The dilution at which each antigen was assayed corresponds to the linear part of the curve after a titration analysis. As a secondary antibody, an affinity-isolated goat anti-human IgG antibody peroxidase-conjugated (Biosource, New York, USA) at dilution 1:2,000 was used. The reaction was incubated for 1 h at 37°C. After washing, the reaction was developed using orthophenylenediamine for 5 minutes in the dark at room temperature. The reaction was stopped by addition of 2N sulfuric acid and the absorbance was measured at 492 nm. Serum samples were assayed in triplicate and at two dilutions. Positive and negative control sera were included in all plates. The cut-off values represent the mean value of the reactivity of a pool of sera from healthy donors (sensitivity) or from patients with autoimmune or infectious diseases other than Chagas disease (specificity), plus two standard deviations.

### Statistical analysis

Statistical analyses were performed using the SPSS statistical package version 15.0 (Spss Inc., Chicago Illinois). The Mann Withney U test was used to carry out comparisons among groups of patients. The Wilcoxon test was used to study post-treatment evolution. Statistical significance was assigned at a value of p ≤ 0.05. Pearson correlation analyses were used to evaluate the correlation between reactivity levels against KMP11, HSP70, PFR2 and Tgp63. Significant correlation was assigned at a value of p ≤ 0.05.

## Results and Discussion

### Recognition of KMP11, HSP70, PFR2, Tgp63, and STcA by sera from chagasic patients

**Figure 1:**
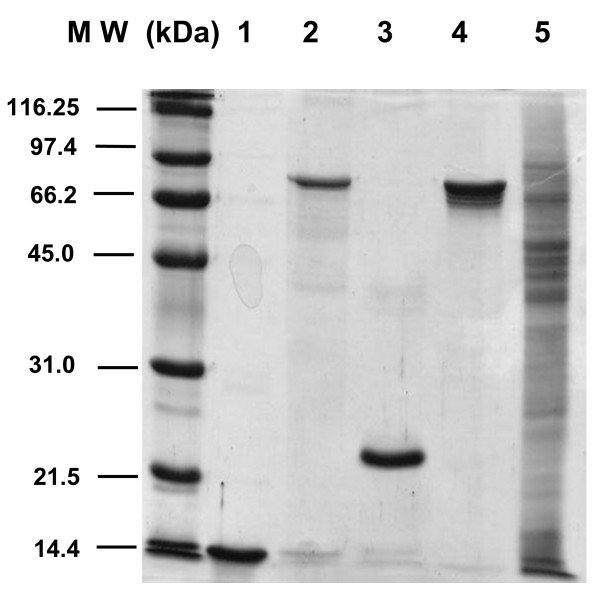
**Purified recombinant antigens and soluble proteins from *T. cruzi***. The KMP11 (lane 1), HSP70 (lane 2), Tgp63 (lane 3), PFR2 (lane 4) recombinant proteins and the total protein lysate, S*Tc*A (lane 5) were resolved in a 12% SDS-PAGE gel and visualized by Coomassie brilliant blue staining.

**Figure 2:**
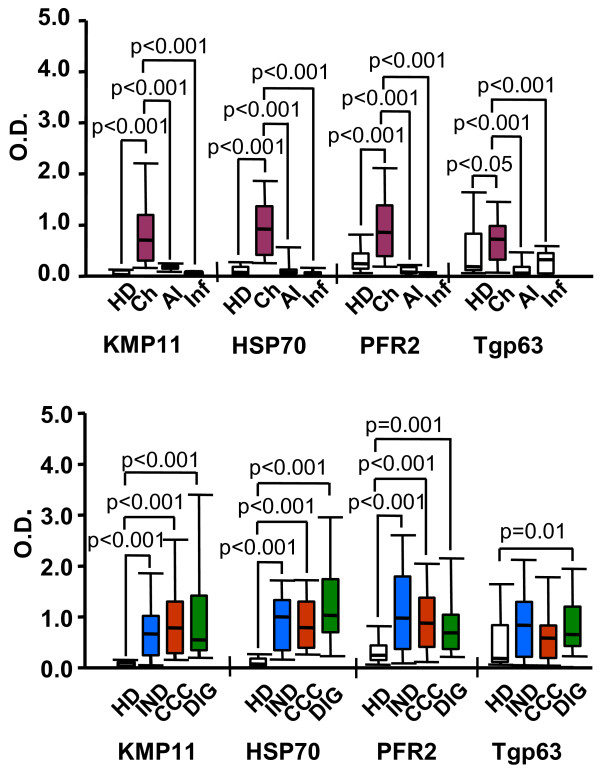
**IgG antibodies specific to KMP11, HSP70, PFR2 and Tgp63 parasite recombinant antigens**. The level of IgG antibodies was measured by ELISA. Data are expressed as optical density (O.D.) values. Statistically significant differences (p < 0.05) are indicated. O.D. values of the percentage of patients comprised between 25% and 75% are represented as vertical boxes and the corresponding medians as a horizontal line inside the boxes. Error bars represent 10% and 90% intervals **(A) **Sera from 22 healthy donors (HD), 46 non-treated chagasic patients (Ch), 15 patients with autoimmune disease (AI) and 24 patients with an infectious disease other than Chagas disease (Inf). **(B) **Sera from 22 healthy donors (HD), 15 indeterminate (IND), 16 cardiac (CCC) and 15 digestive (DIG) chagasic patients.

The *T. cruzi *KMP11, HSP70, and PFR2 proteins, and the *L. infantum *Tgp63 fragment (Figure 1, lanes 1-4) were selected among several recombinant proteins because in previous reports [26,30,31] and in preliminary laboratory screening they proved to be immunodominant antigens in chagasic patients. For comparison, a *T. cruzi *total soluble protein extract (S*Tc*A) (Figure 1, lane 5) was also included in the study. As shown in Figure 2A, the KMP11, HSP70, PFR2 and Tgp63 recombinant proteins are recognized with statistical significance by the sera from chagasic patients (n = 46) relative to that from healthy donors (HD, n = 22). Furthermore, these antigens do not react with sera from patients with autoimmune diseases (SLE, celiac disease and rheumatoid arthritis) (AI, n = 15) or patients suffering from tuberculosis, leprosy or malaria (Inf, n = 24). However, it is worth mentioning that the sera from patients with leishmaniosis recognize Tgp63 and KMP11 [30,32]. Thus, the specificity and sensitivity is 85% and 90% for KMP11, 85% and 90% for HSP70, 92% and 75% for PFR2 and 70% and 30% for Tgp63.

When the reactivity data were analyzed in relation to the clinical stage of Chagas' disease, it was observed (Figure 2B) that the sera from IND, CCC and DIG groups recognize with statistical significance the KMP11, HSP70, PFR2 recombinant proteins when compared with healthy donors (HD). These antigens are recognized by sera from chagasic patients regardless of the stage of the illness. In DIG patients, the reactivity level against Tgp63 was also higher than that detected in the sera from HD. The results presented suggest that these antigens could be used to detect *T. cruzi *infection in any clinical form of Chagas disease. It has been recently described that the sera from CCC and IND chagasic patients living in endemic areas recognize the *T. cruzi *KMP11 and *Trypanosoma rangeli *HSP70 [33].

In order to determine whether there is any correlation between the reactivity detected in the sera from each one of the chagasic patient against the antigens tested, a paired correlation analysis was carried out. A statistically significant correlation was observed between reactivity against KMP11 and HSP70, with values of r = 0.518 (p = 0.048) in CCC patients and r = 0,632 (p < 0.02) in patients with digestive clinical manifestations. Moreover, a statistically significant correlation was also observed in DIG patients in their response against KMP11 and PFR2, with values r = 0.719 (p < = 0.005). No correlation was observed in the IND patients between any of the antigens under study. This differential reactivity might be a consequence of a particular and differentiated exposure of each one of the antigens to the host immune system due to their specific location in the parasite [27,34] or, alternatively, to the ability of the immune system to recognize antigens depending on the stage of the illness. Tarleton's group has recently described the recognition of a panel of 16 *T. cruzi *proteins by sera from Chagas disease patients living in endemic areas, reporting that the antigens recognition pattern is specific for each serum [23].

### Changes in antigen-specific responses after patient' benznidazole treatment

**Figure 3:**
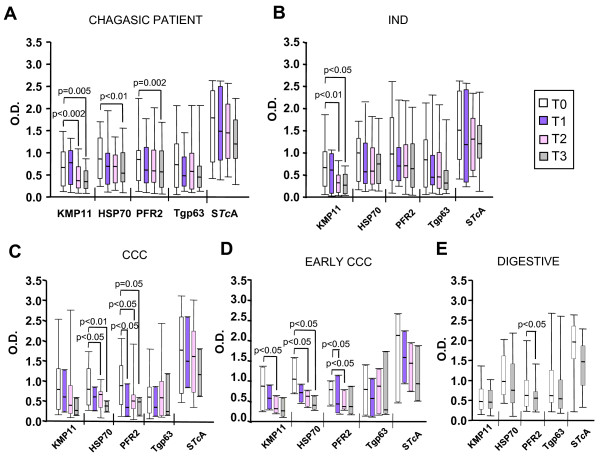
**Effect of benznidazole treatment in antigen recognition**. The reactivity of sera from 25 patients with Chagas disease **(A)**, 15 patients in the indeterminate (IND) stage **(B)**, 10 in the chronic and cardiac phase (CCC) **(C)**, 8 patients in the early chronic cardiac stage of the disease (G1 and G2) **(D) **and 10 digestive chagasic patients **(E) **were assayed by ELISA against KMP11, HSP70, PFR2, Tgp63 and S*Tc*A before benznidazole treatment (T0), and 3 (T1), 6 (T2), and 9 (T3) months post-treatment for **A, B, C **and **D **and 9 (T3) months post-treatment for **E**. Data are expressed as optical density (O.D.) values. The data are the result of a statistical analysis of the median of the differences in antigen-specific recognition that were separately analyzed in each treated patient at the different post-treatment times relative to T0. The p values were obtained by the Wilcoxon matched pair test and significant differences (p < 0.05) are indicated.

To address the effect of a 60-day course of benznidazole treatment in the pattern of recognition of the above mentioned antigens, serum samples from a total of 35 chagasic patients (15 IND, 10 CCC and 10 DIG) were analyzed before (T0) and after benznidazole administration at regular time intervals (3, 6 and 9 months) T1, T2 and T3, respectively. The follow-up of the serological response could only be carried out in 35 patients out of the 46 initially enrolled because 11 of them changed their residence and moved to another city or returned to their countries. Interestingly, a short term decrease in reactivity against KMP11, HSP70 and PFR2 was observed post-treatment. The decrease in reactivity against KMP11 occurred six months and nine months against PFR2 and HSP70 (Figure 3A). The drop in reactivity remained or continued decreasing during the post-treatment follow-up period (two years). A further decrease in reactivity was observed in 67%, 50% and 34% of the patients for KMP11, HSP70 and PFR2, respectively (data not shown). No statistically significant drop in reactivity was observed against S*Tc*A and Tgp63. Thus, at a humoral response level, analysis of the reactivity against these antigens is likely to be a useful tool for evaluating the influence of benznidazole treatment. One of the challenges in Chagas disease is the lack of early markers in the cure or progression of the disease after parasitic treatment. The use of whole parasite lysates or specific recombinant antigen mixture hardly ever provides conclusive results in short- or medium-term follow-up of Chagas patients under treatment [9,22,35]. PCR could be useful in identifying treatment failure in patients with positive detection of *T. cruzi *DNA in their blood stream but does not prove treatment success since even repeated negative PCR results do not necessarily indicate parasitological cure [3]. In fact there is no direct correlation between a negative PCR result and *T. cruzi *infection [3].

When the reactivity against the recombinant antigens was analyzed taking into consideration the stage of the disease of the treated patients, variations in reactivity against the individual antigens were observed. Thus in patients grouped as being in the indeterminate stage, a statistically significant decrease in reactivity against KMP11 was only observed 6 months post-treatment (Figure 3B). However, in chronic cardiac patients, a statistically significant decrease in reactivity was detected against HSP70 and PFR2 (Figure 3C). This fall in anti-PFR2 and anti-HSP70 specific antibody level was detected 3 and 6 months post-treatment, respectively. When the reactivity of the sera from chronic cardiac patients was evaluated in detail, a decrease in the recognition of KMP11, in addition to PFR2 and HSP70, was observed in patients in the early phase of the cardiomyopathy (G1 and G2 stages) (Figure 3D). Nonetheless, a statistically significant drop in reactivity against the antigens was not observed in the chronic cardiac chagasic patients in the advanced stage of the disease (G3). In DIG patients, a statistically significant decrease in reactivity against PFR2 was only observed 9 months post-treatment (Figure 3E). These data indicate that the short-term decrease in recognition of single and specific parasite antigens observed after benznidazole treatment are differentially regulated depending on the clinical stage of the disease. A statistically significant drop in reactivity against the S*Tc*A was not observed in any patient group (Figure 3A-E).

The observed decrease in the antigen specific humoral responses in sera of patients following benznidazole treatment could be interpreted as the result of a balance between several effects in which the immune competence of the infected host is a factor to be considered. Most likely, benznidazole treatment could reduce the parasite load and thus, also reduce the amount of antigen necessary to activate an antibody response as has been recently suggested [36]. In fact, there are already data indicating that benznidazole treatment in experimentally infected mice induces a decrease in the percentage of circulating parasites [37,38]. It is also probable that the antibodies generated against KMP11, PFR2 and HSP70 antigens of the parasite have a short half-life and therefore, are likely to be influenced by the treatment. Although further studies testing sera from a higher number of chagasic patients from endemic areas could be necessary to draw conclusive data for an immunological clinical follow-up, the results presented here show that the detection of reactivity against different and specific antigens may be useful for monitoring the effectiveness of benznidazole treatment.

## Conclusions

This study shows that benznidazol treatment in chagasic patients alters the humoral response pattern against specific antigens in a sickness status dependent manner. This paper describes a useful serological marker system for monitoring the effectiveness of drug treatment in Chagas disease, and a tool that may well allow early detection of therapeutic failure.

## Competing interests

The authors declare that they have no competing interests.

## Authors' contributions

Conception and design of experiments: CM, MCT, MS and specially MCL. Execution of experiments: MJP, BC, CM, MCT and specially, AF. Data Analyses: AF, CM, MCT, MCL. Contribution of reagents/materials/analysis tools: AF, MJP, CM, MCT, EP, BC, JG, MS, MCL. Wrote the paper: MCT and MCL, and the initial draft AF and CM. All authors have read and approved the final manuscript.

## Pre-publication history

The pre-publication history for this paper can be accessed here:

http://www.biomedcentral.com/1471-2334/11/206/prepub
